# Studying the Evolving Knowledge of Adverse Drug Reactions in Order to Facilitate the Rational Use of Medicines in Paediatric Patients

**DOI:** 10.3390/healthcare7020055

**Published:** 2019-04-02

**Authors:** Kristina Star, Imti Choonara

**Affiliations:** 1 Research Section, Uppsala Monitoring Centre, S-751 40 Uppsala, Sweden; 2 Academic Division of Child Health, University of Nottingham, Derby DE22 3DT, UK; Imti.Choonara@nottingham.ac.uk

**Keywords:** adverse drug reactions, drug toxicity, rational prescribing, pharmacovigilance

## Abstract

Pharmacovigilance, which is the science and activities relating to the detection, assessment, understanding and prevention of adverse effects or any other possible drug-related problems, generates knowledge to facilitate the rational use of medicines. When a medicine is first marketed, there is limited information on adverse drug reactions (ADRs), especially in paediatrics, where medicines are less likely to have been extensively studied. Knowledge in drug safety is built up over time when more (in number, and more heterogeneous) patients are treated than were studied in the randomised controlled trials preceding the marketing of a medicine. Previously not recognised ADRs are often initially described in case reports and case series. Prospective cohort studies are useful in determining the incidence and risk factors of common ADRs. Case series and pharmacovigilance reporting systems have been useful in identifying previously unknown uncommon ADRs and risk factors for specific ADRs. This brief review provides examples that illustrate how various study designs and data sources contribute to the evolving knowledge of ADRs that is essential to help develop guidelines and improve the rational use of medicines.

## 1. Introduction

All medicines have potential side effects. Studies have shown that up to around 2 in 10 children in hospital experience adverse drug reactions (ADRs) [1,2], and that at least 1 in 500 children in the community will experience an ADR each year [3].

Most commonly, ADRs are predictable, dose-related and are caused by a medicine’s pharmacological action. ADRs can also be uncommon or rare, unpredictable and occur at commonly used therapeutic doses [4]. ADRs can be induced by drug–drug interactions, misuse, medication errors, or be associated with risk factors such as genetic susceptibility, age, gender or pre-existing medical history.

Before a medicine is considered safe and effective, it has been tested on animals (not in developing or new born animals) and patients in randomized clinical trials (RCTs). The RCTs use homogenous groups of patients restricted in number (from a few hundred to about 3000 [5]), treatment duration, and disease severity. The elderly and children are most often excluded from these premarketing RCTs unless the drug is specifically developed for these populations. Therefore, when medicines are new, or start to be used in new populations, knowledge on ADRs is limited. This situation puts a heavy responsibility on healthcare professionals to stay vigilant and report any suspected ADR when prescribing or administering medicines. Regulatory authorities, manufacturers and academia study the toxicity of a medicine during its complete life cycle and rely on collaborations with healthcare professionals and patients.

Pharmacovigilance is “the science and activities relating to the detection, assessment, understanding and prevention of adverse effects or any other possible drug-related problems” [6]. The core task in pharmacovigilance is to contribute knowledge that can be used for the safe and rational use of medicines. There are many different ways of studying drug safety. Later phase RCTs are the gold standard for studying the efficacy of medicines. They are, however, less useful for studying uncommon ADRs as they are often under-powered in numbers, and ADRs from RCTs are poorly reported [7]. Apart from experimental studies like RCTs, observational studies can be used to study ADRs [8]. Prospective cohort studies are useful for providing information on the incidence and risk factors of common ADRs [2,9,10]. This is because a group of patients are followed over time and, thereby, the denominator is known, which allows one to calculate the incidence. They are, however, poor at detecting rare ADRs which can only be determined with very large numbers of patients. Other limitations with cohort studies are biases relating to subject selection and loss to follow-up [8]. To compare groups with, and without, a specific outcome, such as a specific type of ADR, case-control studies can be used. However, case-control studies are usually retrospective and can suffer from various types of biases, such as sampling (referring to both cases and controls) and recall biases [8]. 

Clinical observation, case reports and case series are useful for identifying possible new ADRs and can sometimes be the only source of information for understanding their nature. To enable sharing suspicions of ADRs, apart from publications in scientific journals, a globally unified collection of spontaneous reports to pharmacovigilance reporting systems was set up following the thalidomide tragedy in the 1960s [11]. Information on suspected ADRs has been collated since 1968, when ten nations joined to form the World Health Organization (WHO) Programme for International Drug Monitoring (PIDM). Healthcare professionals, patients and pharmaceutical manufacturers report to national pharmacovigilance centres who forward data to VigiBase, the WHO global database of individual case safety reports maintained by Uppsala Monitoring Centre (UMC) [12,13]. In December 2018, 134 nations were members of the WHO PIDM and shared over 18 million reports collected since the start of the programme. More than half of these reports have been transferred to VigiBase during the past five years, with a current growth of about two million reports per year.

The knowledge compiled from these different sources is used to guide healthcare professionals and patients in their efforts to mitigate risks from medication use. 

The aims of this brief review are to illustrate different methods of studying drug safety and to explore how they have been applied to enhance safe prescribing in paediatric patients. Several examples of recognised drug toxicity in children will be used. This paper is not a comprehensive review of drug safety in children, but rather focuses on examples of ADRs that illustrate the route from suspicion of the ADR to recognition by clinicians, and on how various study designs and data sources contribute with increasing knowledge.

## 2. Chloramphenicol

An early example from 1959 is when the antibiotic chloramphenicol was reported in a case series of three neonates who developed the grey baby syndrome (abdominal distension, vomiting, cyanosis and cardiovascular collapse) [14]. 

The following year, a pharmacokinetic study showed that neonates had impaired glucuronidation of chloramphenicol [15]. This study suggested that drug metabolism may be impaired in the neonatal period and that, in view of this, lower doses should be used (Table 1). Subsequent studies have shown that glucuronidation is significantly impaired in neonates [16]. 

In the US, the product information for intravenous chloramphenicol states that full-term neonates up to two weeks old, or paediatric patients with immature metabolic function, should receive half the daily paediatric dose divided into four doses to avoid serious ADRs. The blood levels should also be carefully followed. The product is restricted in use because of other known serious ADRs, such as blood dyscrasias, seen in all ages; hence “chloramphenicol must not be used when less potentially dangerous agents will be effective” [17]. 

Chloramphenicol is also used as topical treatment for eye or ear infections. In the UK, some of these topical products are indicated for children of two years and over, and other products do not have an age restriction; however, guidance suggests that it may be necessary to adjust the dose when used in newborn babies [18].

## 3. Sulphonamides

The use of sulphonamides in premature neonates was reported to be associated with both an increased mortality and kernicterus [19]. This was actually identified in an RCT. In the mid-1950s, antibiotics were recommended to prevent serious infection in premature neonates; however, there was no guidance as to which treatment regimen should be used. Silverman et al. initiated an RCT investigating mortality and morbidity in 193 low birth weight neonates randomized to either oxytetracycline or penicillin/sulfisoxazole [19]. The study finding of increased mortality and kernicterus was unexpected as clinical trials in the 1950s preceded the era of power calculations in order to determine the appropriate sample size. Clinical trials are now powered to look at efficacy, but not usually powered to detect toxicity. A few years later, a study demonstrated that sulphonamides are highly protein bound and that, therefore, they displace bilirubin from albumin [20]. This results in an increase in the free fraction of bilirubin and can cause kernicterus in a pre-term neonate (Table 1). Subsequently, it was recognised that one needs to avoid highly protein bound medicines in the neonatal period in order to minimise the risk of kernicterus. Sulphonamide products in the UK are contraindicated in premature babies and full-term infants during the first six weeks of life, except for the treatment and prophylaxis of pneumocystis pneumonia, and should be avoided in late pregnancy and by mothers who are nursing babies at risk for hyperbilirubinaemia [21].

## 4. Salicylates

In 1963, a case series of 21 children with multi-organ failure including encephalopathy, liver failure and renal impairment was reported [22]. The children all presented to a single hospital in Australia over a period of 11 years. Seventeen of the 21 children died. In 1965, a letter postulated a possible link between Reye’s syndrome and salicylates [23]. It was a clinical observation by one individual based on cases seen in a single hospital. It was not until a case-control study in 1980 that the use of salicylate was confirmed to be associated with Reye’s syndrome [24]. This case-control study involved seven children hospitalised for Reye’s syndrome and 16 controls from schools in one state in the USA. The mechanism of salicylates causing Reye’s syndrome was not established. However, regulatory agencies subsequently recommended avoiding the use of salicylates as an antipyretic/analgesic in children under the age of 12 years in many countries [25]. Risk minimising action can be taken even when the mechanism of the ADR cannot be explained. A review of a national pharmacovigilance database for suspected ADRs reported four fatalities in association with the use of salicylates in children between the ages of 12 and 16 years who developed Reye’s syndrome [26] (Table 2). Subsequently, salicylates were no longer recommended in children under 16 years in the UK [27] as an analgesic/antipyretic. When it comes to the mechanism, there is evidence that aspirin seems to trigger the development of Reye’s syndrome in patients with fatty acid oxidation disorders [28]. 

## 5. Propofol

Propofol was developed as an anaesthetic agent. It was subsequently used as a sedative in intensive care settings. In 1992, a case series of five children with upper respiratory tract infections aged between four weeks and six years, who developed metabolic acidosis, bradycardia, heart failure following the use of propofol, was reported [29]. Sadly, the five children died. Several years later, a larger case series of 18 children showed that the toxicity was likely to be dose-related [30]. It was recognised that high dose (>4 mg/kg/h) and long duration (more than 48 h) were likely to significantly increase the risk of the development of propofol infusion syndrome. In Europe, propofol is now contraindicated as a sedative in ventilated children [31]. Despite this, propofol continues to be used in critically ill children [32].

## 6. Antiepileptic Drugs

Epilepsy is the most common neurological disorder in children. Sodium valproate is the most frequently prescribed antiepileptic drug although there is increasing utilisation of newer antiepileptic drugs such as levetiracetam [33]. Case reports of hepatotoxicity following the use of valproic acid were first reported in 1979 [34]. In 1987, a retrospective review of 37 cases of fatal hepatotoxicity associated with valproate therapy was published [35]. This retrospective review identified children under the age of three years receiving polytherapy as being at greatest risk of developing liver failure. Additionally, the presence of developmental delay was also a risk factor. This case series review suggested avoiding the use of valproic acid in these children at greatest risk. A subsequent analysis of VigiBase reviewed 156 fatalities in children in association with hepatotoxicity and valproic acid [36]. This study again suggested polytherapy to be a risk factor but also identified that the risk appeared greatest in young children aged six years and under. 

Several antiepileptic drugs can be associated with Stevens–Johnson syndrome and toxic epidermal necrolysis. Lamotrigine has been reported to be associated with the development of Stevens–Johnson syndrome. It was initially described in case reports [37]. Co-medication with valproic acid has been described in individual case reports. A case series of 10 children who developed a serious skin reaction to lamotrigine (including five possible cases of Stevens–Johnson syndrome) identified co-medication with valproic acid and high dose/rapid dose escalation as risk factors [38]. In 2000, the lamotrigine dosage regimen for children was revised in the UK to reduce the risk of this ADR [39]. A review of paediatric case reports of Stevens–Johnson syndrome and toxic epidermal necrolysis in association with lamotrigine in VigiBase identified 486 reports. Co-medication with valproic acid was reported in 207 of the 486 cases (43%), which was significantly more than for cases with these co-medications reported without skin reactions [40].

## 7. Medications Used for Attention-Deficit Hyperactivity Disorder

Attention-deficit hyperactivity disorder (ADHD) is a widespread disorder that can be managed by medication such as methylphenidate. In 2005, the regulatory agency of Health Canada signalled 20 cases of sudden death reported worldwide (14 were children) for a stimulant combination product containing dexamphetamine and amphetamine that had been used for patients with ADHD. Health Canada initially withdrew the product but subsequently allowed it back on the market after a warning had been added to the product information that the product should not be used in patients with structural cardiac abnormalities [41]. Another signal, issued by UMC in 2005, highlighted serious cardiac effects after the use of the newly marketed non-stimulant ADHD medicine atomoxetine. The report described 38 cases with prolonged QT interval, seven with cardiac arrest and five cases with ventricular tachycardia [42]. In 2006, Health Canada updated the labelling for stimulant and non-stimulant ADHD drugs regarding very rare cardiac-related adverse events [41]. 

A case-control study in 2009 reported an association between stimulant use and sudden death in adolescents [43]. The US FDA reminded their prescribers to screen children for heart disease before prescribing these medications. In 2011, a large retrospective cohort study was published that showed no evidence of an increased risk of cardiovascular events [44]. A systematic review and meta-analysis, however, found effects of methylphenidate on systolic blood pressure and both atomoxetine and amphetamines increased systolic and diastolic blood pressure as well as heart rate [45]. These medicines are centrally acting sympathomimetics and increase noradrenergic transmission, so a plausible mechanism for cardiac reactions exists [45].

The current product information for methylphenidate, dexamfetamine and atomoxetine extensively describes how to manage the risks of these potential ADRs. Treatment is contraindicated in patients with pre-existing cardiovascular disorders such as structural cardiac abnormalities and life-threatening arrhythmias; so careful pre-treatment screening for cardiovascular status should be conducted and the patient’s ongoing cardiovascular status should be monitored during treatment [46,47,48]. 

## 8. Discussion

Our knowledge on medicines in children has increased significantly since the 1950s. Toxicity due to impaired metabolism or bilirubin displacement is, therefore, less likely to occur today as medicines are now more likely to be formally studied in children before being used widely. It is important, however, to recognise that medicines are less extensively studied in children than in adults. Additionally, many medicines are studied in healthy older children and not the sickest and youngest. One, therefore, needs to be aware of the risk of toxicity when using new medicines, and when a child is exposed to a medicine for the first time. Clinical trials are beneficial in evaluating the efficacy of medicines. However, they rarely detect uncommon ADRs. Knowledge generated from prospective cohort studies can provide information on the incidence and characteristics of ADRs; but they are often restricted to specific settings and lengths of time, which limits their ability to capture uncommon ADRs and ADRs occurring in specific patient subgroups. Despite over 6000 admissions, as in one prospective study [2], many severe reactions were low in numbers when related to a specific drug. Hence, knowledge generation of uncommon reactions can be limited in these types of studies.

Published case reports or case series and reviews from pharmacovigilance reporting systems can guide prescribers in relation to the nature of harm associated with medications (Table 2). These sources can be used to identify possible new ADRs in paediatric patients such as acute renal impairment in association with levetiracetam [49]. Case series and pharmacovigilance reporting systems are useful for identifying possible risk factors for severe but uncommon ADRs [40]. Well-documented case series can give insights into the nature of ADRs such as when the event is expected to occur in relation to the commencement of treatment with a medicine and inform about the chronology of signs and symptoms leading up to the diagnosis of an ADR, as well as the outcome and time to recovery. The major disadvantage with analysing global pharmacovigilance reports is that the risk cannot be measured because the data lacks information on the denominators and numerators. The number of treated patients (with or without a reaction) is unknown as well as the number of patients with a certain reaction (in relation to a drug or not). Other limitations with spontaneous reports are data quality problems, underreporting, and reporting biases due to intensively monitored drugs or media attention. One of the major advantages with pharmacovigilance reporting systems is the possibility to capture knowledge about any type of reaction, setting (hospital, out-patient care), medicine or medication use (off-label use, prescription, or over-the-counter), or patient (handicap or multi-sick), over the whole time a drug is available and used. 

## 9. Conclusions

This brief review describes how knowledge about the safety of medicines is built up over time. To illustrate this, we have used some common historical examples and mainstream study designs and data sources. However, we have not included examples based on electronic health records or registries that can contain valuable information on ADRs. In the examples presented, we have referenced national product information; but it should be stressed that license agreements for drugs can differ between national medicines regulatory agencies and that product information changes over time. 

Sharing information and experiences in the context of paediatric medicines safety is imperative to build knowledge on ADRs and its risk factors to help in the development of guidelines to facilitate rational prescribing and use in children. 

## Figures and Tables

**Table 1:** Drug toxicity in neonates.

Drug	Adverse Reaction	Type of Evidence and Year
Chloramphenicol	Grey baby syndrome	Case series (1959) [14] Pharmacokinetic study (1960) [15]
Sulphonamides	Kernicterus	Randomized Controlled Trial (1956) [19] Protein binding study (1964) [20]

**Table 2:** Drug toxicity in children.

Drug	Adverse Reaction	Type of Evidence and Year
Salicylates	Reye’s syndrome	Case series (1963) [22] Letter postulating link to salicylates (1965) [23] Case-control (1980) [24] National pharmacovigilance report review (2002) [26]
Propofol	Multi-organ failure Propofol infusion syndrome	Case series (1992) [29] Case series (1998) [30]
Valproic acid	Hepatotoxicity	Case report (1979) [34] Case series (1987) [35] Global pharmacovigilance report review (2014) [36]
Lamotrigine	Stevens–Johnson syndrome	Case report (1999) [37] Case series (1999) [38] Global pharmacovigilance report review (2017) [40]
Psychostimulants/non-stimulants	Cardiac-related events	Case reports (2005) [41,42] Case-control (2009) [43] Meta-Analysis of trials (2017) [45]
